# Chloroform Inhalation

**Published:** 1847-12

**Authors:** Buckminster Brown

**Affiliations:** Boston


8. *Chloroform Inhalation.*—Mr. Editor,—A letter received by the last steamer from Dr. W. J. Little, Surgeon of the London Hospital, and the well-known founder of the Royal Orthopedic Institution, contains, with other interesting medical intelligence, important information concerning the employment of a new agent for producing insensibility during surgical operations, and in obstetric cases. This new agent, which it appears is exciting much attention among the English Surgeons, is chloroform, a substance first discovered by Soubeiran in France, and at nearly the same time by Liebig in Germany. Its introduction, however, to the medical public as an anæsthetic agent, is due to Professor Simpson, of the Edinburgh University, who has published the result of his experience. The following extracts from the letter to which I have referred, and from Professor Simpson's pamphlet, for which I am also indebted to Dr. Little, contain the most important items relative to this subject. Dr. L. states in his letter that he has tried and has witnessed the trial of chloroform, and from his experience thus far feels prepared to say that "the facility and rapidity with which the effect is produced, the absence of annoyance to the inhalers and bystanders, the tranquil manner in which a person who has inhaled recovers from the state of insensibility, and the length of time he remains insensible, even after inhaling the chloroform only thirty to thirty-five seconds, give it prominent advantages over ether." Among other advantages which Dr. Simpson affirms this compound to possess over ether, are—"its having an agreeable fragrant, fruit-like odor, and a saccharine taste; that a much less quantity of chloroform is required to produce the anæsthetic effect, usually from 100 to 120 drops of chloroform only being sufficient, and with some patients much less. I have seen," he says, "a strong person rendered completely insensible by six or seven inspirations of 40 drops of the liquid. Its action is more rapid and complete, and generally more persistent. I have almost always seen from ten to twenty inspirations suffice. Hence the time of the surgeon is saved, and the preliminary stage of excitement is practically abolished. Its perfume is not unpleasant, but the reverse, and the odor of it does not remain for any length of time obstinately attached to the clothes of the attendant, or exhaling in a disagreeable form from the lungs of the patient. No sickness, vomiting, headache, salivation, uneasiness of chest in any of the cases. Being required in much less quantity, it is much more portable than sulph. ether." The above extracts I send you without comment, as of course it is too early to form an opinion, and will only add that I have employed a chemist to prepare a quantity of this article, with which it is my intention to institute a succession of experiments.

*Boston, Dec.* 27, 1847. BUCKMINSTER BROWN.

—*Ibid.*

